# Gauging the Awareness of Physicians in Saudi Arabia Regarding Risk Factors for Thyroid Cancer

**DOI:** 10.7759/cureus.53747

**Published:** 2024-02-06

**Authors:** Saad M Alqahtani, Musaed Rayzah, Riyaz A. Shaik, Mansour K Alzahrani, Yousef Alalawi, Sahar Alnefaie, Mohammad S. Ahmad

**Affiliations:** 1 Department of Surgery, College of Medicine, Majmaah University, Al-Majmaah, SAU; 2 Department of Family and Community Medicine, College of Medicine, Majmaah University, Al-Majmaah, SAU; 3 Department of Surgery, King Salman Armed Forces Hospital, Tabuk, SAU; 4 Department of Surgery, College of Medicine, Taif University, Taif, SAU

**Keywords:** thyroid cancer, saudi arabia, physicians, obesity, knowledge

## Abstract

Background

The prevalence of obesity and thyroid cancer (TC) is increasing worldwide, and obesity is a risk factor for TC.

Objectives

This study aimed to elucidate physicians’ awareness of obesity as a risk factor for TC.

Materials and methods

A cross-sectional, self-report online questionnaire was distributed to physicians in all regions of Saudi Arabia. The questionnaire comprised sociodemographic data and questions concerning the risk factors for TC, including obesity.

Results

A total of 310 physicians participated in this study. Of the respondents, 35.8% (*n* = 111) were aged 30-40 years, 40.6% (*n* = 126) were board certified, and 52.3% (*n *= 162) had >10 years of experience. Only 36.8% (*n *= 114) of respondents were familiar with the relationship between obesity and TC risk (*P* < 0.001). In terms of knowledge of obesity as a risk factor for TC, a significant difference was observed for the following sociodemographic characteristics: sex, educational attainment, and years of experience. A significant difference was also observed with awareness of other risk factors for TC.

Conclusions

In light of the limited awareness of the correlation between obesity and TC, the most effective approach to address these misconceptions would be to implement diverse and ongoing medical education initiatives.

## Introduction

Obesity and cancer are two major public health concerns in most developing and developed countries [1]. Thyroid cancer (TC) ranks as the most frequent endocrine malignancy worldwide, including in Saudi Arabia [2]. Moreover, there has been a parallel increase in the global prevalence of obesity, which is considered to be a risk factor for TC development [3].

The higher rates of TC are assumed to be a result of overdiagnosis secondary to the increased availability of neck ultrasonography. However, some experts believe that overdiagnosis is not the only possible explanation, and other potentially modifiable factors are being investigated. One well-supported theory is the correlation between a higher body mass index (BMI) and the likelihood of developing TC [4].

A history of radiation, family history of TC, chemicals, lifestyle habits (such as smoking and physical inactivity), and comorbidities (such as diabetes mellitus, insulin resistance, obesity, iodine deficiency, and metabolic syndrome) are risk factors for TC [5-7], with obesity being the second most common avoidable and modifiable risk factor after smoking [7].

Globally, the prevalence of obesity has experienced an almost threefold increase since 1975; in 2016, 39% of individuals aged ≥18 years were classified as overweight and 13% as obese [8]. Furthermore, 65% of the world’s population lives in areas where overweight and obesity are more likely to cause disease and death than malnutrition [9,10]. Obesity is associated with various comorbidities, including obstructive sleep apnea, infertility, cardiovascular diseases, hypercholesterolemia, hypertension, diabetes mellitus, depression, and cancer-related conditions, including lung, brain, stomach, breast, pancreas, endometrial, esophageal, colorectal, liver, and thyroid malignancies [11]. Notably, a five-point increase in BMI is associated with a 30% increase in the risk of developing TC, and a 0.1-point increase in the waist-to-hip ratio is associated with a 14% increased risk of TC [12]. Although obesity and differentiated TC, particularly papillary thyroid carcinoma (PTC), have a well-established positive association [13], the precise underlying mechanisms remain unclear. However, several significant factors have been postulated to account for this relationship, including inflammation, adipokines, insulin resistance, thyroid hormones, oxidative stress, and sex hormones [9].

The high incidence of overweight and obesity in Saudi Arabia is a cause of considerable concern; 50% of Saudi Arabia’s adult population is overweight, and one in every five individuals is obese [8]. Thus, considering its contribution to disability and mortality, controlling obesity will lead to better quality of life, the prevention of other obesity-related conditions, less demand on healthcare facilities, and the prioritization of treatment for other prevalent diseases.

Therefore, obesity remains a major issue affecting all societies, including Saudi Arabia. As the increasing prevalence of obesity may contribute to the observed increase in PTC, it is imperative that physicians familiarize themselves with this potential risk factor; however, to our knowledge, no studies have investigated this to date. Thus, the aim of this survey-based study was to assess the awareness of physicians in Saudi Arabia regarding obesity as a risk factor for TC, with the intention of promoting a healthy population.

## Materials and methods

This cross-sectional self-report online questionnaire was distributed to physicians across all regions of Saudi Arabia. A total of 310 physicians participated in this study, and the data were collected in May, June, and July 2023. Physicians from all regions of Saudi Arabia were included, but endocrine surgeons, surgical oncologists, endocrinologists, medical oncologists, and ear, nose, and throat surgeons were excluded. The study was conducted in accordance with the most updated version of the Declaration of Helsinki and approved by the Research Ethics Committee of Majmaah University (Ethics number: MUREC-Apr.3/COM-2023/13-1, approval date 3/4/2023). Informed consent was obtained from all participants.

Participants were selected via purposive sampling, which is a form of non-probability sampling. The questionnaire was developed after reviewing the literature, and a sample of five participants was selected for pretesting the modified draft, which was then finalized for the main study. The validity of the questionnaire was ascertained through expert review and pilot testing and further examined using five neutral respondents who were not involved in the study. This rigorous testing process provided a well-structured questionnaire to collect reliable data. The final version was constructed using Google Forms (Google, Mountain View, CA, USA) and distributed via an open link.

The form began with an explicit agreement to participate and subsequently proceeded with a series of 12 questions. The questionnaire comprised two parts: (1) demographic data, qualifications, years of experience, specialty, and residency (working area); and (2) knowledge regarding the difference between obesity and overweight, how to calculate BMI, and whether obesity is linked to cancer in general and TC in particular, as well as questions about awareness of other thyroid risk factors, including iodine deficiency, goiters, a family history of TC, previous radiation exposure, a high level of thyroid-stimulating hormone (TSH), and the presence of inflammation (i.e., Hashimoto’s thyroiditis). The questions regarding knowledge could be answered with “yes,” “no,” or “I don’t know.”

The participants were able to review and modify their responses prior to finalizing and submitting the questionnaire. To ensure survey completeness, responses could only be submitted once all items were answered. Moreover, all data were secured and protected.

Data were entered into Excel (Microsoft, Redmond, WA, USA) and analyzed using IBM SPSS Statistics for Windows, Version 20.0 (Released 2011; IBM Corp; Armonk, New York, United States). Sociodemographic features are expressed as frequencies and percentages. The participants’ awareness of each factor was assessed using the chi-square goodness of fit test, and associations between sociodemographic characteristics and awareness of obesity as a risk factor for TC were analyzed using the chi-square test. A threshold of *P* < 0.05 was set for statistical significance.

## Results

Sociodemographic features

Table 1 presents an overview of the participants’ sociodemographic characteristics.

**Table 1:** The sociodemographic features of the respondents.

Characteristics	Category	Number (%)
Age (in years)	<30	55 (17.7)
	30–40	111 (35.8)
	41–50	93 (30.0)
	>50	51 (16.5)
Sex	Male	227 (73.2)
	Female	83 (26.8)
Qualification	MBBS	82 (26.5)
	Masters	72 (23.2)
	PhD	30 (9.7)
	Board certified	126 (40.6)
Nationality	Saudi	155 (50.0)
	Non-Saudi	155 (50.0)
Specialty	General surgeon	93 (30.0)
	General practitioner	31 (10.0)
	Family medicine	72 (23.2)
	Community medicine	6 (1.9)
	Obstetrics and gynecology	4 (1.3)
	Internal medicine	40 (12.9)
	Other	64 (20.6)
Years of experience	<5	73 (23.5)
	5–10	75 (24.2)
	>10	162 (52.3)
Working area (region)	North	52 (16.8)
	East	29 (9.4)
	Central	95 (30.6)
	West	40 (12.9)
	South	94 (30.3)

Of the 310 physicians who participated in this study, most were male (*n* = 227, 73.2%), and 35.8% (*n* = 111) fell within the age range of 30-40 years. Additionally, 40.6% (*n* = 126) possessed board certifications, and 50.0% (*n* = 155) were of Saudi descent. The majority of respondents had a specialization in general surgery (*n* = 93, 30.0%), with family medicine physicians representing the second largest group (*n* = 72, 23.2%). In contrast, obstetrics and gynecology had the lowest number of respondents (*n* = 4, 1.3%). Notably, general practitioners accounted for 10.0% (*n* = 31) of the participants.

A significant proportion of the participants reported having amassed a professional tenure >10 years (52.3%, *n* = 162), whereas a comparatively smaller percentage indicated having <5 years of experience (23.5%, *n* = 73). The central and southern regions of Saudi Arabia displayed a comparable representation of the highest proportion, constituting approximately 30% each (*n* = 95 and 94, respectively), whereas the eastern region was the smallest at 9.4% (*n* = 29).

Overall awareness

Table 2 depicts the scope of knowledge regarding cancer and obesity.

**Table 2:** Knowledge levels among the participants. TSH: thyroid-stimulating hormone; TC: thyroid cancer; BMI: body mass index. Data are presented as *n* (%) and statistically significant if *P* < 0.05.

Knowledge	Yes	No	I don’t know	*P*-value
General Information				
Is there any difference between overweight and obesity?	304 (98.1%)	4 (1.3%)	2 (0.6%)	<0.001
Do you know how to calculate BMI?	306 (98.7%)	2 (0.6%)	2 (0.6%)	<0.001
Is obesity a modifiable and preventable disease?	307 (99.0%)	3 (1.0%)	0 (0.0%)	<0.001
Is obesity associated with cancer?	217 (70.0%)	47 (15.2%)	46 (14.8%)	<0.001
Knowledge of other risk factors for TC				
Iodine deficiency	160 (51.6%)	87 (28.1%)	63 (20.3%)	<0.001
Goiter	213 (68.7%)	74 (23.9%)	23 (7.4%)	<0.001
Family history of TC	295 (95.2%)	11 (3.5%)	4 (1.3%)	<0.001
Radiation exposure	300 (96.8%)	5 (1.6%)	5 (1.6%)	<0.001
High TSH levels	117 (37.7%)	117 (37.7%)	76 (24.5%)	0.004
Presence of inflammation (Hashimoto's thyroiditis)	205 (66.1%)	64 (20.6%)	41 (13.2%)	<0.001
Is obesity a risk factor for TC?	114 (36.8%)	46 (14.8%)	150 (48.4%)	<0.001
If Yes, for which type of TC?	Well-differentiated TC	Medullary TC	I don’t know	
	31 (27.2%)	20 (17.5%)	63 (55.3%)	<0.001

Over 98% of participants were familiar with the difference between overweight and obesity, BMI calculations, and obesity as a modifiable and preventable disease (*n* = 304, 306, and 307, respectively). However, only 70% of participants (*n* = 217) accurately identified obesity as being associated with cancer, with 15.2% (*n* = 47) recognizing no association and 14.8% (*n* = 46) indicating uncertainty. These findings were statistically significant (*P* < 0.001).

Knowledge of risk factors for TC

For the questions pertaining to the presence of a familial background of TC and exposure to radiation as potential risk factors for TC, most respondents (95.2% and 96.8%, respectively) provided accurate responses. Approximately 50% of participants (*n* = 160) demonstrated awareness of the correlation between iodine deficiency and the risk of TC, and approximately two-thirds exhibited awareness regarding both the risk of goiters and the presence of inflammation (*n* = 213 and 205, respectively). All of these risk factors showed statistically significant associations (*P* < 0.001), as shown in Table 2. Notably, 117 (37.7%) participants correctly identified high TSH as a risk factor for TC, whereas an equal number responded with “no” and 24.5% (*n* = 76) were uncertain (*P* = 0.004).

Remarkably, only 36.8% (*n* = 114) of participants provided an accurate response regarding the correlation between obesity and the risk of TC (*P* < 0.001). Of the respondents who provided accurate responses, 27.2% (*n* = 31) were familiar with the correlation between obesity and well-differentiated TC, although the majority were uncertain regarding the specific types of TC associated with obesity (*P* < 0.001).

Table 3 shows the correlation between knowledge of obesity as a risk factor for TC and sociodemographic features, with statistically significant associations with sex, educational attainment, and years of professional experience. In terms of qualifications, physicians with PhDs achieved the highest rate of accuracy (*n* = 17, 56.7%), whereas physicians who had board certifications exhibited the lowest rate of accuracy (*n* = 34, 27.0%). Furthermore, individuals with >10 years of expertise achieved the highest accuracy (*n* = 73, 45.1%) when compared with respondents with fewer years of experience.

**Table 3:** Correlation between awareness of obesity as a TC risk factor and sociodemographic features. TC: thyroid cancer. Data are presented as *n* (%) and statistically significant if *P* < 0.05. The *P*-values were generated using the chi-squared test.

Sociodemographic features		Is obesity a risk factor for TC?			*P*-value
		Yes	No	I don’t know	
Age (in years)	<30	14 (25.5%)	11 (20.0%)	30 (54.5%)	0.082
	30–40	35 (31.5%)	18 (16.2%)	58 (52.3%)	
	41–50	38 (40.9%)	12 (12.9%)	43 (46.2%)	
	>50	27 (52.9%)	5 (9.8%)	19 (37.3%)	
Sex	Male	88 (38.8%)	39 (17.2%)	100 (44.1%)	0.025
	Female	26 (31.3%)	7 (8.4%)	50 (60.2%)	
Qualification	MBBS	30 (36.6%)	13 (15.9%)	39 (47.6%)	0.032
	Masters	33 (45.8%)	11 (15.3%)	28 (38.9%)	
	PhD	17 (56.7%)	3 (10.0%)	10 (33.3%)	
	Board certified	34 (27.0%)	19 (15.1%)	73 (57.9%)	
Specialty	General surgeon	36 (38.7%)	13 (14.0%)	44 (47.3%)	0.362
	General practitioner	12 (38.7%)	5 (16.1%)	14 (45.2%)	
	Family medicine	26 (36.1%)	10 (13.9%)	36 (50.0%)	
	Community medicine	5 (83.3%)	0 (0.0%)	1 (16.7%)	
	Obstetrics and gynecology	2 (50.0%)	0 (0.0%)	2 (50.0%)	
	Internal medicine	14 (35.0%)	3 (7.5%)	23 (57.5%)	
	Other	19 (29.7%)	15 (23.4%)	30 (46.9%)	
Years of experience	<5	20 (27.4%)	16 (21.9%)	37 (50.7%)	0.017
	5–10	21 (28.0%)	12 (16.0%)	42 (56.0%)	
	>10	73 (45.1%)	18 (11.1%)	71 (43.8%)	
Working area (region)	North	22 (42.3%)	8 (15.4%)	22 (42.3%)	0.086
	East	9 (31.0%)	10 (34.5%)	10 (34.5%)	
	Central	37 (38.9%)	14 (14.7%)	44 (46.3%)	
	West	12 (30.0%)	4 (10.0%)	24 (60.0%)	
	South	34 (36.2%)	10 (10.6%)	50 (53.2%)	

## Discussion

The global incidence of both obesity and PTC is on the rise, and a well-supported theory for this is the association between a high BMI and the likelihood of developing TC [4]. A recently published study showed that the incidence of TC in Saudi Arabia markedly increased in both sexes between 1990 and 2019; specifically, the incidence increased 22-fold in men and 15-fold in women [2]. The etiology of this condition remains uncertain; however, a number of potential factors have been identified, including a familial predisposition to TC, tobacco use, inadequate iodine intake, obesity, previous exposure to radiation (especially in childhood), high TSH levels, Hashimoto’s thyroiditis, and elevated leptin levels [2,14,15]. Awareness of these risk factors can help monitor and track patients who are at high risk of TC.

From 1975 to 2016, the prevalence of obesity and overweight in Saudi Arabia substantially increased, with a higher prevalence in women than in men. In 2019, the majority of adults in Saudi Arabia (three out of five) were overweight or obese [8]. Furthermore, the prevalence in children has significantly increased by nearly threefold within the last 40 years. In 2016, the prevalence of obesity or overweight among children and adolescents in Saudi Arabia was found to be 33%, which is double the global average of 18%. This increasing trend is anticipated to continue as the population matures. Furthermore, the phenomenon of the sex gap in the rates of overweight and obesity saw a reversal over the period of 1999-2002, with men now exhibiting a higher prevalence of both conditions than women [8]. These data highlight the significant issue that the Saudi community is currently confronting.

In accordance with Vision 2030, Saudi Arabia’s comprehensive plan to diminish its reliance on oil, broaden its economic base, and enhance public service sectors such as healthcare and education, the nation has set forth the aim of achieving a 3% decline in obesity rates by 2030 [8]. To accomplish this objective, developing interventions that leverage current data and evidence is critical, with a particular focus on prioritizing preventive measures over treatment strategies. Additionally, collaboration across many sectors, both within and beyond healthcare, is essential [8]. As leaders of the healthcare system, physicians have the vital role of implementing such strategies and measures. Therefore, the rationale of this study was to assess physicians’ awareness regarding the association between TC and obesity.

The World Cancer Research Fund and the International Agency for Research on Cancer have both acknowledged the substantial evidence linking overweight and obesity to the development, treatment, and progression of various types of cancer, including TCs, leukemia, non-Hodgkin’s lymphoma, esophageal adenocarcinoma, endometrial cancer, colon cancer, postmenopausal breast cancer, prostate cancer, pancreatic cancer, liver cancer, and myeloma [9]; however, these do not appear to be equally affected by obesity [16]. Notably, obesity was determined to be the cause of 3.6% of all malignancies worldwide, accounting for 481,000 new cases a year, and was responsible for 4.5 million deaths worldwide in 2013 [16].

Obesity is defined as the abnormal accumulation of fat tissue in the body, constituting >20% of the total body weight in men and >25% in women. Adipocytes produce and trigger the release of cytokines, growth factors, and many other hormones that interfere with the regulation of cell growth and survival [16]. Obesity is widely recognized as a leading cause of carcinogenesis and is linked to elevated rates of cancer occurrence and progression [3]. A comprehensive review conducted in 2010 estimated that approximately 20% of cancer cases may be attributed to excessive weight [17]. Kitahara et al. found that in 2015 approximately one in every six cases of PTC detected in individuals aged ≥60 years may be attributed to the presence of overweight and obesity [3]. In addition, they demonstrated that between 1995 and 2015 overweight and obesity increased the incidence of PTC [3]. Moreover, a meta-analysis of 31 studies found that obesity is associated with a greater risk of TC in women [4]. However, the mechanisms underlying the correlation between weight gain and TC are not fully understood. Elevated leptin levels, oxidative stress, inflammation, enhanced insulin resistance, high TSH levels, and increased aromatase activity have been postulated as plausible explanations [7].

Notably, maintaining a healthy weight is associated with a lower risk of TC in both sexes, implying that weight control programs promote cancer prevention [4]. Furthermore, a substantial correlation was found between weight gain and an increased TC risk, whereas weight loss was associated with a decreased TC risk [4]. A prospective cohort study revealed that those classified as overweight or obese exhibited a higher susceptibility to TC than those with a normal weight [18]. Similarly, Kitahara et al. demonstrated a positive correlation between an elevated BMI and an increased susceptibility to TC [19], and a study of 351,402 participants found that a higher BMI was closely correlated with the risk of TC [1]. Additionally, another study found that lifestyle changes may minimize the prevalence of TC caused by obesity [13].

Interestingly, Funk et al. reported that physicians have a poor understanding of obesity treatment alternatives [20]. Furthermore, according to a cross-sectional survey, 70% of individuals with obesity were not diagnosed, and 63% received no guidance on weight loss, dietary changes, or exercise [21]. Similarly, Bleich et al. found that the majority of individuals with obesity did not obtain a diagnosis or weight-related counseling regarding weight loss, diet, or exercise [22].

The results of our study revealed that approximately 48.4% of physicians (*n* = 150) responded “I don’t know” when asked about the causal relationship between obesity and TC. In contrast, only 36.8% (*n* = 114) provided the correct response. To address this issue, monthly medical education meetings should be held to discuss common community diseases (such as obesity), their consequences, and their prevention. Such measures are called primary prevention and refer to reducing and controlling risk factors to prevent overt illnesses in healthy people [23].

Of the respondents who provided accurate responses regarding the association between obesity and TC, 29 physicians (27.2%) indicated that well-differentiated TC is the most commonly observed type among individuals who are obese, whereas 20 (17.5%) chose medullary TC as their answer. The prevalence of different TC types, including papillary, follicular, and anaplastic, has been widely acknowledged in individuals with obesity. Conversely, obesity is associated with a diminished likelihood of developing medullary TC, indicating a possible reliance on tumor characteristics and histological specificity [7].

Screening and further assessment of obesity can be performed using BMI measurements [10]. Despite the difficulty of thyroid palpation in patients with obesity and the positive association between obesity and PTC, there is insufficient evidence to support routine neck ultrasonography in the obese population [13,24]. Nevertheless, relying solely on palpation is inadequate in patients with obesity and should be accompanied by neck ultrasound. Consequently, some investigators have suggested personalized approaches to better monitor and treat these patients [13].

Our study has many strengths, including being the first to examine the level of awareness among physicians regarding obesity as a risk factor for TC. Furthermore, it is distinguished by its comprehensive inclusion of all regions of Saudi Arabia and its sufficiently large sample size. However, we must acknowledge certain limitations, including the use of an online questionnaire for data collection and reliance on self-reporting, both of which have the potential to affect the accuracy of the information obtained. Moreover, a sampling bias may have arisen from the inclusion of only those physicians with a preexisting interest in the subject matter.

## Conclusions

In conclusion, our findings revealed that only 36.8% of physicians (*n* = 114) were aware that obesity is a potential risk factor for TC. Thus, it is imperative to recognize and emphasize the need for continuing medical education for healthcare providers regarding prevalent diseases and their ensuing complications. Moreover, underscoring the significance of healthcare decision-makers in enacting a comprehensive nationwide approach aimed at fostering health consciousness and mitigating the prevalence of obesity is crucial. Therefore, we advocate for the enhancement of collaborative efforts with nonprofit organizations and the promotion of their involvement in community health in conjunction with the government sector. The implementation of lifestyle modifications that promote a decrease in the occurrence of obesity may result in a corresponding reduction in the occurrence of TCs that are influenced by obesity.
